# Unraveling the molecular mechanisms underlying neocortical tau propagation in Alzheimer’s disease to reveal novel insights into clinical heterogeneity

**DOI:** 10.1002/alz.084995

**Published:** 2025-01-03

**Authors:** Anjalika Chongtham, Marissa Farinas, Diede Broekaart, Joon Ho Seo, Aarthi Ramakrishnan, Carolyn W. Zhu, Mary Sano, Li Shen, Ana C. Pereira

**Affiliations:** ^1^ Icahn School of Medicine at Mount Sinai, New York, NY USA; ^2^ James J. Peters VA Medical Center, Bronx, NY USA; ^3^ Ronald M. Loeb Center for Alzheimer’s Disease, New York, NY USA

## Abstract

**Background:**

Heterogeneity in the progression of clinical dementia poses a significant challenge, impeding the effectiveness of current therapies for Alzheimer’s disease (AD). To decipher the molecular mechanisms governing heterogeneity in AD progression that remains a critical knowledge gap precluding rational therapeutic design, we investigated the biochemical and biophysical properties of tau present in the inferior temporal gyrus (ITG) and prefrontal cortex (PFC) brain regions of AD patients who had varying disease progression rates. To explore gene expression changes in the ITG which are associated with tau pathology and cognitive decline, we used RNA sequencing for molecular characterization of patients displaying tau and clinical heterogeneity.

**Method:**

Postmortem brain samples from the ITG and PFC of AD (n = 20) and control (n = 8) subjects were used to evaluate the biochemical features of abnormal tau species, specifically those that may influence its propagation and best predict AD progression. Further, we investigated molecular heterogeneity in AD with RNA‐seq analyses of ITG tissues with differential seeding potential. The present study integrates tau biochemistry with transcriptional alterations to elucidate the molecular mechanisms of cognitive decline in AD, which is critical for advancing future therapeutic interventions and development of personalized treatments.

**Result:**

Biochemical analysis of human postmortem ITG and PFC tissues revealed individual variability in tau seeding, which correlated with cognitive decline, particularly in the ITG, a region known for promoting accelerated tau propagation. Specific hyperphosphorylated high and, intriguingly, low‐molecular‐weight tau (p‐T217, T231, S396 and S396/S404‐tau) and their isoforms (3R and 4R‐tau) are likely mediators of seeding and cognitive decline. Additionally, RNA‐seq transcriptomic analyses showed that cognitive decline during AD progression could be related to tau‐mediated failure of neural mechanisms in the ITG, particularly a loss of neural and synaptic plasticity and increased neuroinflammation.

**Conclusion:**

These findings provide further insights into multiple molecular mechanisms potentially involved in disease progression, highlight targets for early intervention, and improve patient subtyping, which is critical for developing precision medicines.

